# Supplementary material for: Heparin-based hydrogel scaffolding alters the transcriptomic profile and increases the chemoresistance of MDA-MB-231 triple-negative breast cancer cells
Source: Biomater Sci. 2020 Feb 13;8(10):2786–96. doi: 10.1039/c9bm01481k (PMC7497406; doi:10.1039/c9bm01481k)
Supplement: Supplementary file 2 [file BM-008-C9BM01481K-s002.zip › Supplementary File 4/HP-BhydrogelvControl/Pathways/my_analysis.Gsea.1545201079907/gsea_report_for_CONTROL_1545201079907.html]

Report for CONTROL 1545201079907 [GSEA]

| GS  follow link to MSigDB | GS DETAILS | SIZE | ES | NES | NOM p-val | FDR q-val | FWER p-val | RANK AT MAX | LEADING EDGE || 1 | HALLMARK\_OXIDATIVE\_PHOSPHORYLATION | Details ... | 194 | -0.66 | -3.16 | 0.000 | 0.000 | 0.000 | 3003 | tags=58%, list=16%, signal=68% |
| 2 | HALLMARK\_PROTEIN\_SECRETION | Details ... | 94 | -0.67 | -2.89 | 0.000 | 0.000 | 0.000 | 3205 | tags=60%, list=17%, signal=71% |
| 3 | HALLMARK\_MYC\_TARGETS\_V1 | Details ... | 189 | -0.56 | -2.64 | 0.000 | 0.000 | 0.000 | 5073 | tags=60%, list=26%, signal=80% |
| 4 | HALLMARK\_MTORC1\_SIGNALING | Details ... | 190 | -0.52 | -2.44 | 0.000 | 0.000 | 0.000 | 4168 | tags=47%, list=22%, signal=59% |
| 5 | HALLMARK\_FATTY\_ACID\_METABOLISM | Details ... | 131 | -0.46 | -2.12 | 0.000 | 0.000 | 0.000 | 3898 | tags=47%, list=20%, signal=58% |
| 6 | HALLMARK\_ANDROGEN\_RESPONSE | Details ... | 91 | -0.49 | -2.11 | 0.000 | 0.000 | 0.000 | 3295 | tags=38%, list=17%, signal=46% |
| 7 | HALLMARK\_ADIPOGENESIS | Details ... | 176 | -0.44 | -2.06 | 0.000 | 0.000 | 0.000 | 3321 | tags=40%, list=17%, signal=48% |
| 8 | HALLMARK\_HEME\_METABOLISM | Details ... | 166 | -0.39 | -1.86 | 0.000 | 0.002 | 0.005 | 2965 | tags=30%, list=15%, signal=35% |
| 9 | HALLMARK\_GLYCOLYSIS | Details ... | 171 | -0.38 | -1.81 | 0.000 | 0.003 | 0.009 | 3092 | tags=32%, list=16%, signal=38% |
| 10 | HALLMARK\_REACTIVE\_OXIGEN\_SPECIES\_PATHWAY | Details ... | 44 | -0.45 | -1.66 | 0.009 | 0.009 | 0.029 | 2400 | tags=32%, list=13%, signal=36% |
| 11 | HALLMARK\_COAGULATION | Details ... | 87 | -0.39 | -1.65 | 0.003 | 0.008 | 0.030 | 5106 | tags=46%, list=27%, signal=62% |
| 12 | HALLMARK\_PEROXISOME | Details ... | 85 | -0.40 | -1.65 | 0.000 | 0.008 | 0.030 | 5636 | tags=58%, list=29%, signal=81% |
| 13 | HALLMARK\_COMPLEMENT | Details ... | 141 | -0.36 | -1.64 | 0.000 | 0.007 | 0.031 | 3889 | tags=33%, list=20%, signal=41% |
| 14 | HALLMARK\_E2F\_TARGETS | Details ... | 192 | -0.34 | -1.64 | 0.000 | 0.007 | 0.031 | 6398 | tags=47%, list=33%, signal=70% |
| 15 | HALLMARK\_INTERFERON\_ALPHA\_RESPONSE | Details ... | 91 | -0.38 | -1.64 | 0.000 | 0.006 | 0.031 | 5477 | tags=49%, list=29%, signal=69% |
| 16 | HALLMARK\_APOPTOSIS | Details ... | 140 | -0.34 | -1.58 | 0.000 | 0.012 | 0.066 | 3791 | tags=31%, list=20%, signal=38% |
| 17 | HALLMARK\_PANCREAS\_BETA\_CELLS | Details ... | 18 | -0.52 | -1.57 | 0.031 | 0.012 | 0.069 | 1840 | tags=33%, list=10%, signal=37% |
| 18 | HALLMARK\_DNA\_REPAIR | Details ... | 139 | -0.33 | -1.52 | 0.000 | 0.019 | 0.108 | 3716 | tags=32%, list=19%, signal=39% |
| 19 | HALLMARK\_HYPOXIA | Details ... | 163 | -0.32 | -1.51 | 0.000 | 0.019 | 0.114 | 4222 | tags=35%, list=22%, signal=44% |
| 20 | HALLMARK\_INTERFERON\_GAMMA\_RESPONSE | Details ... | 167 | -0.32 | -1.48 | 0.000 | 0.020 | 0.131 | 4619 | tags=35%, list=24%, signal=45% |
| 21 | HALLMARK\_CHOLESTEROL\_HOMEOSTASIS | Details ... | 67 | -0.36 | -1.44 | 0.032 | 0.029 | 0.190 | 5190 | tags=48%, list=27%, signal=65% |
| 22 | HALLMARK\_KRAS\_SIGNALING\_UP | Details ... | 144 | -0.29 | -1.34 | 0.019 | 0.064 | 0.395 | 4430 | tags=31%, list=23%, signal=39% |
| 23 | HALLMARK\_ALLOGRAFT\_REJECTION | Details ... | 116 | -0.29 | -1.32 | 0.024 | 0.069 | 0.427 | 3725 | tags=27%, list=19%, signal=33% |
| 24 | HALLMARK\_PI3K\_AKT\_MTOR\_SIGNALING | Details ... | 89 | -0.31 | -1.32 | 0.044 | 0.071 | 0.450 | 3355 | tags=31%, list=17%, signal=38% |
| 25 | HALLMARK\_BILE\_ACID\_METABOLISM | Details ... | 80 | -0.31 | -1.32 | 0.047 | 0.069 | 0.454 | 3540 | tags=30%, list=18%, signal=37% |
| 26 | HALLMARK\_UNFOLDED\_PROTEIN\_RESPONSE | Details ... | 105 | -0.28 | -1.25 | 0.073 | 0.108 | 0.623 | 3262 | tags=26%, list=17%, signal=31% |
| 27 | HALLMARK\_ESTROGEN\_RESPONSE\_LATE | Details ... | 161 | -0.27 | -1.23 | 0.055 | 0.120 | 0.685 | 3255 | tags=24%, list=17%, signal=28% |
| 28 | HALLMARK\_G2M\_CHECKPOINT | Details ... | 190 | -0.26 | -1.23 | 0.027 | 0.121 | 0.697 | 5456 | tags=37%, list=28%, signal=51% |
| 29 | HALLMARK\_IL2\_STAT5\_SIGNALING | Details ... | 155 | -0.25 | -1.18 | 0.089 | 0.166 | 0.807 | 3642 | tags=25%, list=19%, signal=31% |
| 30 | HALLMARK\_XENOBIOTIC\_METABOLISM | Details ... | 151 | -0.25 | -1.17 | 0.115 | 0.173 | 0.830 | 4345 | tags=32%, list=23%, signal=41% |
| 31 | HALLMARK\_IL6\_JAK\_STAT3\_SIGNALING | Details ... | 63 | -0.26 | -1.07 | 0.299 | 0.323 | 0.965 | 4163 | tags=24%, list=22%, signal=30% |
| 32 | HALLMARK\_SPERMATOGENESIS | Details ... | 76 | -0.25 | -1.03 | 0.367 | 0.410 | 0.992 | 2251 | tags=18%, list=12%, signal=21% |
| 33 | HALLMARK\_EPITHELIAL\_MESENCHYMAL\_TRANSITION | Details ... | 150 | -0.21 | -1.00 | 0.462 | 0.485 | 0.994 | 2767 | tags=21%, list=14%, signal=25% |
| 34 | HALLMARK\_UV\_RESPONSE\_UP | Details ... | 131 | -0.21 | -0.97 | 0.529 | 0.541 | 0.999 | 3897 | tags=23%, list=20%, signal=29% |
| 35 | HALLMARK\_INFLAMMATORY\_RESPONSE | Details ... | 130 | -0.16 | -0.73 | 1.000 | 0.966 | 1.000 | 6441 | tags=41%, list=34%, signal=61% |
Table: Gene sets enriched in phenotype **CONTROL (2 samples) **[plain text format]****
